# Fisheries-independent surveys identify critical habitats for young scalloped hammerhead sharks (*Sphyrna lewini*) in the Rewa Delta, Fiji

**DOI:** 10.1038/s41598-017-17152-0

**Published:** 2017-12-08

**Authors:** Amandine D. Marie, Cara Miller, Celso Cawich, Susanna Piovano, Ciro Rico

**Affiliations:** 1 School of Marine Studies, Faculty of Science, Technology and Environment, The University of the South Pacific, Suva, Fiji; 2 School of Science and Technology, University of New England, Armidale, NSW Australia

## Abstract

Sharp declines in numerous shark populations around the world have generated considerable interest in better understanding and characterising their biology, ecology and critical habitats. The scalloped hammerhead shark (SHS, *Sphyrna lewini*) is subject to a multitude of natural and anthropogenic threats that are often exacerbated within the coastal embayments and estuaries used during SHS early life stages. In this study, we describe the temporal and spatial distribution, age class composition, and reproductive biology of SHS in the Rewa Delta (RD), Fiji. A total of 1054 SHS (including 796 tagged individuals; 101 of which were recaptured) were captured from September 2014 to March 2016 in the RD. A majority of the captures in this area were neonates and young-of-the-year (YOY) (99.8%). Significant seasonality in patterns of occurrence of both neonates and YOY individuals suggests a defined parturition period during the austral summer. Between the seven sampling sites in the RD we also found significant differences in SHS neonate catch per unit of effort, and average total length of individuals. According to the data, the RD is likely to represent an important nursery area for SHS up to one year of age.

## Introduction

In 2010, the global fishing mortality of sharks from catches (both reported and unreported) and discards was estimated to be in excess of 1.4 million tonnes^1^. Global landings reported to the Food and Agriculture Organization (FAO) indicated a rise in the capture of sharks from the 1950s to the late 1990s, followed by a slight decline of 7.5% in the 2000s^1^. Despite this slowing of capture rates in more recent years^2^, the vulnerable life history of many sharks – i.e., being long-lived, late to mature sexually, and exhibiting low fecundity – still causes present harvest rates in many places to be unsustainable. A case in point is in the North-West Atlantic where, amid a steep decline in capture rates of both coastal and oceanic shark populations from 1986 to 2000, hammerhead shark species (*Sphyrna* spp.) suffered a decline equivalent to 89% of its former abundance^3^. While sustainable shark fisheries do exist^4,5^, good information about the species’ biology, ecology and habitat use is needed to develop suitable management plans.

Coastal shark species are known to aggregate at discrete sites for mating, parturition, and maturation^6^. Increasing evidence suggests that at least some sharks have natal philopatry to their birth places^7^ has led to new interest in understanding and characterising critical habitats, such as nursery grounds^8^. More specifically, reproductive philopatry has been described in blacktip (*Carcharhinus limbatus*)^9^, bull (*Carcharhinus leucas*)^7,10^, lemon (*Negaprion brevirostris*)^11^, and scalloped hammerhead (*Sphyrna lewini*, hereafter referred to as SHS)^12^ sharks. In all cases, the degree of site fidelity and distance between nurseries may directly affect the level of population subdivision and genetic divergence among regions, as well as the associated population dynamics^9^. Characterisation of critical habitats, such as nursery areas, is therefore vital to understanding shark ecology and in turn informing effective management and conservation planning for shark species.

The SHS is an apex predator species that has a circumglobal distribution in tropical and warm temperate latitudes over both continental and insular shelves, and adjacent deep oceanic waters^13^. Juveniles and adults occupy different habitats. Juveniles are demersal, gregarious and primarily found in coastal areas, estuaries and embayments. Adults are primarily pelagic, semi-oceanic, and solitary, although they sometimes show aggregation behaviour^13^. Viviparous mature females produce 13–31 neonates of 42–55 cm total length per litter after a gestation period of 9–10 months^14^. This species has also been documented to show natal philopatry^12^.

The SHS was declared an endangered species by the International Union for Conservation of Nature (IUCN) Red List in 2007^15^ and is currently listed on Appendix II of the Convention on International Trade in Endangered Species of Wild Fauna and Flora (CITES)^16^. Several SHS populations have been heavily exploited worldwide by both inshore and offshore fisheries^14^. In Fiji waters, hammerhead sharks are captured by the pelagic longline fishery targeting tunas, and are often discarded after finning^17,18^. In coastal waters, SHS have been reported to use up to seven estuarine areas on Fiji’s two largest islands, Viti Levu and Vanua Levu^19,20^. A local ecological knowledge survey conducted by Rasalato *et al*.^19^ documented the regular occurrence of hammerhead sharks in the Rewa Delta (RD). Furthermore, a preliminary survey conducted between February and May 2012 reported the capture of 82 juveniles in the area, suggesting that the RD could serve as an important nursery area for SHS^20^.

This study focused on developing a more detailed understanding of the reproductive biology and critical habitat of the SHS in the RD, Fiji. More specifically, this multi-year study from September 2014 to March 2016 provides an important summary of the following aspects of the SHS population in RD: spatial and temporal patterns in catch per unit effort (CPUE), temporal trends in parturition period, and key morphometric parameters including total length and population growth rates. To conclude, in light of the new insights of SHS ecology in the RD revealed by this study, we discuss salient conservation and management implications for SHS within the study area and proximal waters.

## Results

**Figure 1:**
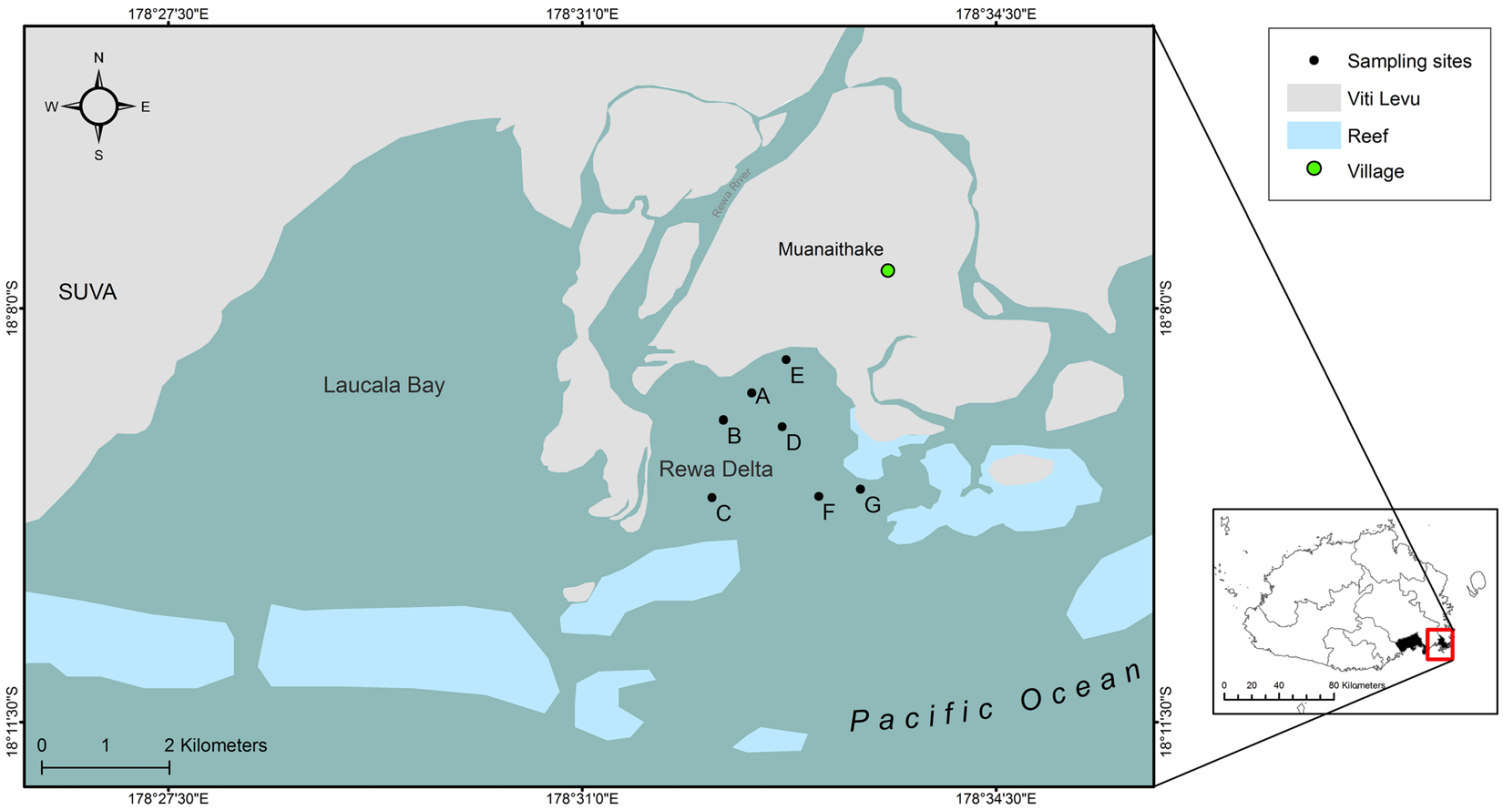
Geographical location of sampling sites (**A**–**G**) in the Rewa Delta, Viti Levu, Fiji. The two maps were created using ArcGis, version 10.2 (https://www.arcgis.com/features/index.html).

A total of 311 gillnet deployments were conducted between September 2014 and March 2016 in the RD, totalling 667.1 standardised hours. A standardised hour was defined as a period of one hour fishing with a 100 m long, 3 m high gill net of 10 cm mesh size. A total of 1054 SHS (including 101 recaptures) were caught during this time (Fig. 1). Of the 953 captured individuals, 796 were tagged and released. Of the 101 recaptured individuals, 89 were captured only once while the remaining 12 were captured up to four times. Furthermore, 93 out of 953 (9.8%) SHS captured during the survey died as a consequence of netting. Of these, 20 were tagged individuals that we recaptured later.

### Population structure dominated by neonates and juveniles

**Figure 2:**
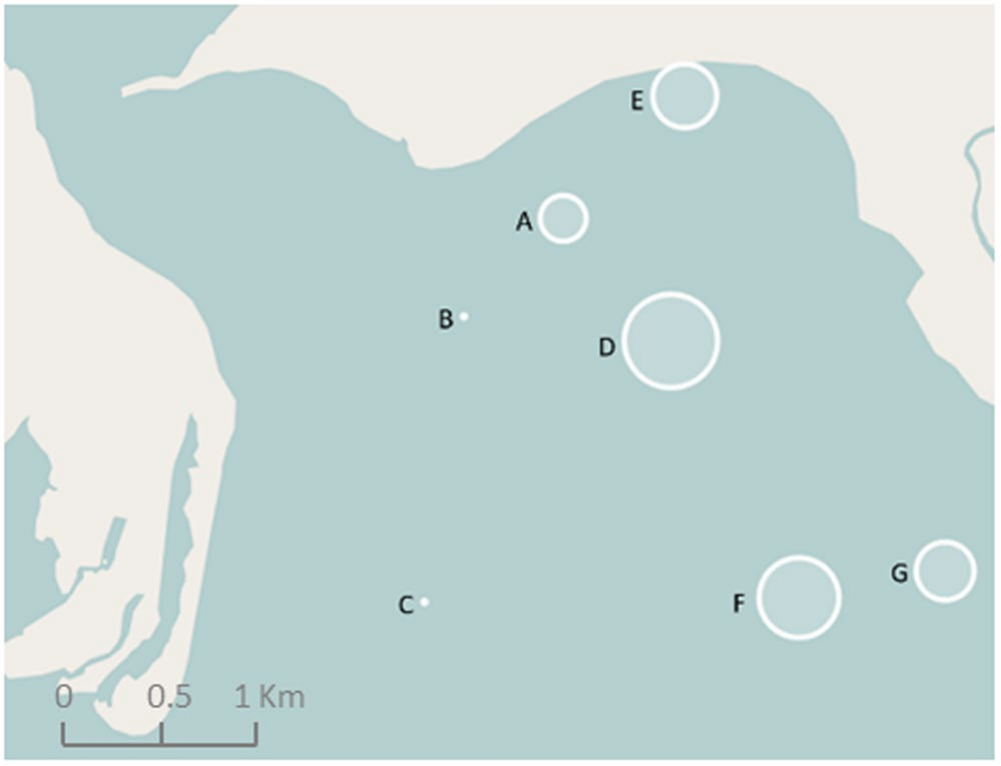
Abundance of scalloped hammerhead sharks, measured through CPUE, on the seven sites in the Rewa Delta (Map data © OpenStreetMap contributors). The size of the circle is indicative of the mean CPUE (i.e. the larger the circle, the higher the CPUE, and the contrary). Image produced using the Leaflet package (version 1.0.1, http://rstudio.github.io/leaflet/) within R statistical software (version 3.3.1)^80^. The R package OpenStreetMap is licensed under a GNU General Public License (GPL-2) (https://cran.r-project.org/web/packages/OpenStreetMap/index.html) and was used to extract map tiles from OpenStreetMap which is licensed on terms of the Open Database License, “ODbL” 1.0. (http://wiki.osmfoundation.org/wiki/Licence).

Only neonates and juveniles (YOY and 1+ year) were caught in the RD throughout the study period. The characterisation of the neonates and juveniles was based on their total length (TL) as well as on the status of their umbilical scars^12^ (Supplementary Table S1). Significant differences in standardised CPUE for all SHS (*P* < 0.016), as well as for neonates only (*P* < 0.032), were observed between the study sites (Table 1; Fig. 2). CPUE for all SHS was significantly different between site C and all other sites except site B, and also between site B and sites D & F (Table 1). In addition, there were different patterns in the spatial distribution of sharks according to their TL (Table 1). Significant differences were found between the following pairwise comparisons: A&F-G, D&F-G and E&F-G, suggesting the segregation of individuals according to size (Table 1). Sites A, D and E appeared to be used by smaller SHS (Supplementary Table S1).

**Table 1:** Pairwise comparisons of CPUE for all sharks, neonates only and the total length of all sharks between sites.

All sharks							
	A	B	C	D	E	F	G
A	*n* = *40*						
B	0.585	*n* = *19*					
C	**0.016**	1	*n* = *24*				
D	0.927	**0.014**	**<0.001**	*n* = *88*			
E	1	0.116	**<0.001**	1	*n* = *57*		
F	0.599	**0.01**	**<0.001**	1	1	*n* = *37*	
G	1	0.312	**0.006**	1	1	0.995	*n* = *33*
**Neonates**							
	**A**	**B**	**C**	**D**	**E**	**F**	**G**
A	*n* = *40*						
B	1	*n* = *19*					
C	1	1	*n* = *24*				
D	**0.023**	0.298	**<0.001**	*n* = *88*			
E	1	1	**0.022**	1	*n* = *57*		
F	1	1	1	**0.032**	1	*n* = *37*	
G	1	1	1	0.079	1	1	*n* = *33*
**Total length of all sharks**							
	**A**	**B**	**C**	**D**	**E**	**F**	**G**
A	*n* = *40*						
B	1	*n* = *19*					
C	1	1	*n* = *24*				
D	1	1	1	*n* = *88*			
E	0.104	1	1	1	*n* = *57*		
F	**<0.001**	0.042	0.099	**<0.001**	**<0.001**	*n* = *37*	
G	**<0.001**	0.07	0.135	**<0.001**	**<0.001**	1	*n* = *33*

Letters A to G refer to the sampling sites (see Figure 1). Numbers below the diagonal are the *P* -values for the test of significant difference in CPUE between two sites, with significant differences shown in bold. Numbers on the diagonal (in italic) indicate the number of net sets for the given site.

### Population structure over time

**Figure 3:**
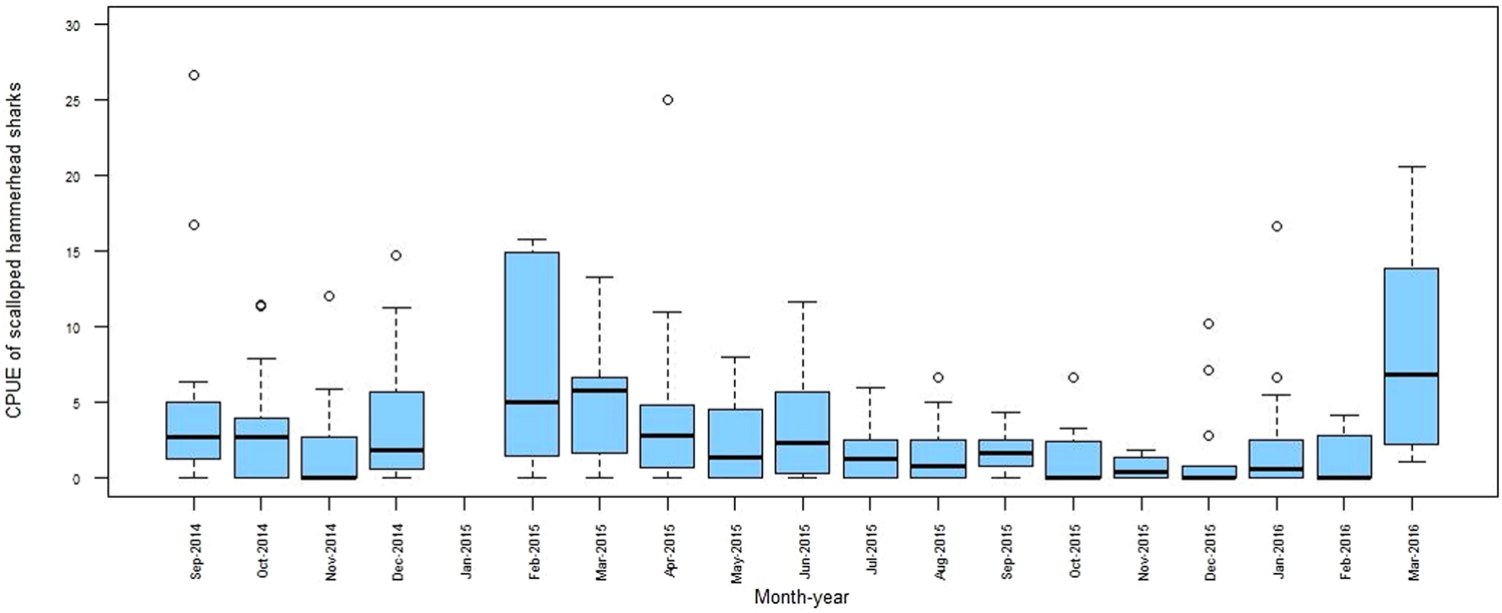
Standardized SHS CPUE for gillnet sampling from September 2014 to March 2016 in the Rewa Delta. The horizontal line in the middle of the box represents the median.

A regular presence of SHS was observed in the RD throughout the 19 months of sampling undertaken for this study (Fig. 3). From September 2014 to March 2016, SHS were caught regularly, although there was some temporal variation in terms of number of sharks caught per month. CPUE per gillnet survey ranged from 0 to 15 SHS/hour (1.81 ± 2.6), with the highest mean CPUE in February 2015 and March 2016, and the lowest in November and December 2015 (Fig. 3). In addition, there is some support for neonate and YOY site fidelity to the RD and its surroundings, as 101 of the 796 tagged sharks (12.7%) were recaptured during the field surveys, and four of them were recaptured more than once for a period ranging between one and 174 days.

### Reproductive biology

**Figure 4:**
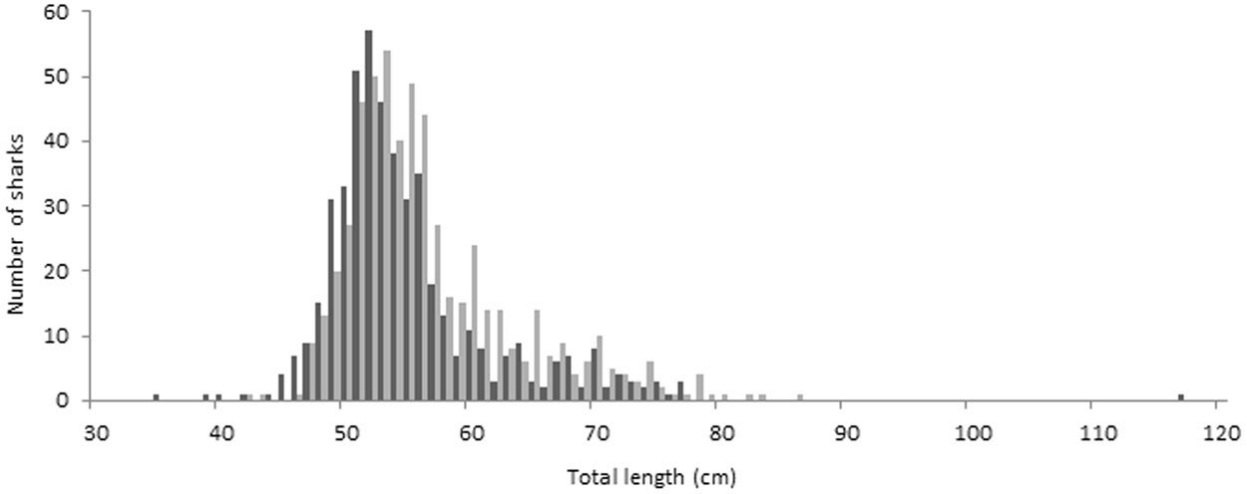
Total length distribution of scalloped hammerhead sharks captured in the Rewa Delta from September 2014 to March 2016. Total length of males and females are represented in pale grey and dark grey respectively.

**Figure 5:**
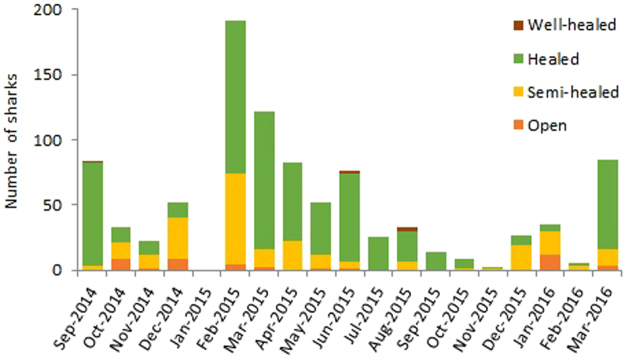
Monthly composition of sampled scalloped hammerhead sharks based on umbilical scars, from September 2014 to March 2016.

**Figure 6:**
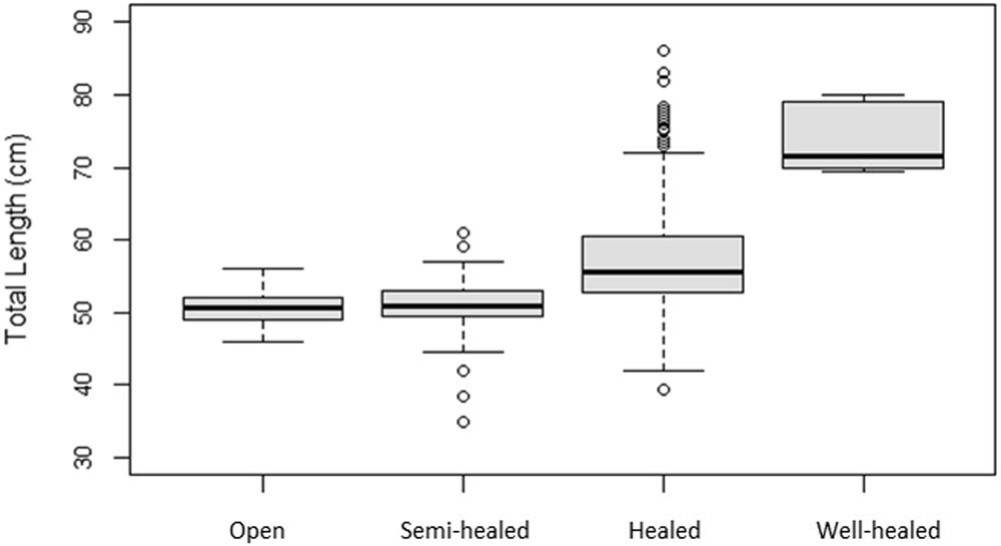
Total length of scalloped hammerhead sharks by umbilical scar category. TL were found to be significantly different in all cases except between individuals with open and semi-healed scars. The horizontal line in the middle of the box represents the median.

The presence of both neonate and YOY SHS was observed in the RD throughout the year. From specimen length distribution, the presence of at least three age classes (i.e. neonates, YOY and 1+ year) was inferred (Fig. 4). Based on the average TL of individuals caught in each sampling site, we found significant differences in the number of sharks of each age class utilising the area throughout the year (Table 1). The results showed that 4.6% and 23.5% of the observed SHS had opened and semi-healed umbilical scars at time of capture, respectively. Chi-square testing of the association between month and umbilical scar status was highly significant (χ^2^, df = 34, *P* < 0.001) (Table 2). Results also showed higher than expected prevalence of open and semi-healed scars from October 2014 to February 2015, and then again from December 2015 to January 2016, suggesting that these may be key birth times for SHS in the RD (Fig. 5). TL of individuals were found to be significantly different between the four umbilical scar status categories, except between individuals with open and semi-healed scars (*P* = 0.057) (Fig. 6). The higher than expected number of SHS with healed umbilical scars between these periods (i.e., March 2015 – September 2015, Fig. 5) supports the proposed seasonality of parturition described above.

**Table 2:** Comparison of the proportion of sharks by different umbilical scar categories per month. A chi-square test indicated that there were significant associations between these two variables (*P* < 0.001). Standardized chi-square Pearson residuals were then used to detect significant scar-month combinations using the following criteria: (i) values less than −2 suggest that the number of observed sharks was lower than expected (bold), (ii) values greater than +2 suggest that the number of observed sharks was higher than expected (italic).

	Open	Semi-healed	Healed
Sep-2014	**−2.040**	**−4.333**	*5.037*
Oct-2014	*8.717*	0.832	**−4.840**
Nov-2014	0.956	*2.788*	**−3.075**
Dec-2014	*4.684*	*5.745*	**−7.599**
Jan-2015	—	—	—
Feb-2015	**−**1.900	*4.445*	**−3.309**
Mar-2015	**−**1.800	**−3.735**	*4.362*
Apr-2015	**−2.140**	0.419	0.598
May-2015	**−**1.030	−1.342	1.745
Jun-2015	−1.760	**−4.220**	*4.798*
Jul-2015	−1.360	**−3.431**	*3.868*
Aug-2015	−1.450	0.425	0.273
Sep-2015	−0.990	**−2.501**	*2.819*
Oct-2015	−0.700	−1.010	1.276
Nov-2015	−0.310	0.886	−0.692
Dec-2015	−1.130	*5.577*	**−4.736**
Jan-2016	*9.054*	*3.598*	**−7.607**
Feb-2016	−0.490	1.932	−1.595
Mar-2016	−0.550	−1.749	1.905

YOY composed the majority of the SHS sampled during the study period (71.9%). YOY were observed in all months and were the only age class captured in July and September 2015 (Fig. 5). Overall, TL ranged from a minimum of 46.0 cm to a maximum of 116.5 cm (Fig. 4; Supplementary Table S1). Values from published literature^21^ suggest that the longest observed male in this study (TL = 86 cm) was at least one year old and longest female (TL = 116.5 cm) was between two and three years old. A summary table of minimum, maximum, and median TL for sexes, scar status and sites reveals patterns in these parameters (Supplementary Table S1).

Using the TL data of each individual marked and recaptured at least once, we were able to estimate the growth rate over the period between first capture and subsequent recaptures. Thus, males (n = 26) and females (n = 11) recaptured between one and five months after being tagged grew a monthly average TL of 2.21 cm ± 1.45 cm and 2.90 cm ± 2.85 cm, respectively. The remaining 64 individuals recaptured were caught for the second time within a week and no differences in size were observed.

## Discussion

This study demonstrated year-round use of the RD by SHS – primarily by neonates and YOY (99.8%). However, we noted both spatial and temporal fluctuations in abundance throughout 19 months of monitoring. More specifically, within the RD we found significant differences in CPUE between the seven sampling sites. These differences are likely linked to the environmental properties of these habitats (see below for further discussion). This study also revealed a clear signal of parturition in the warm and wet months of the austral summer. Such evidence is critical for developing and strengthening shark conservation and management measures within the RD.

Neonates and YOY of coastal shark species are expected to use more sheltered areas to reduce their vulnerability to predation and compensate for their limited foraging skills^12,22^. The data strongly supported this characterisation of habitat use in respect to age status, with almost 99% of the 1046 SHS individuals being categorised as either YOY or neonates (as length-at-age estimates from Branstetter^21^ and Duncan and Holland^12^). Based on the state of their umbilical scar, as well as their TL, which ranged from 46 cm to 56 cm (average of 50.7 cm ± 2.4 cm), we estimate that these individuals were probably two weeks old^12^. A similar range of TL at this age was reported in eastern Australia^23^ (46.5 cm to 56.3 cm) and in the north-western Gulf of Mexico^21^ (46 cm to 60 cm). Using mark-recapture, the monthly growth rate of recaptured individuals in the RD measuring between 48.0 cm and 69.5 cm at the first capture was estimated to be 2.2 cm ± 1.5 cm for males (N = 26) and 2.9 cm ± 2.8 cm for females (N = 11). This growth rate is slower than the one reported by Duncan and Holland^12^ (mean growth rate of 9.6 cm for the first year) and also slower than found in captive SHS (11 cm growth rate for SHS from birth to approximately 100 days old), which may be due to the amount and frequency with which sharks were fed^24^. Although the growth estimates are lower than both published reports, the implications of such a difference can only be explored hypothetically, given the small sample size that was used in this case. Nevertheless, a few potential reasons can be formulated in order to explain these results. Firstly, this slow growth rate provides some evidence that the RD does not contain adequate prey volume to support its SHS aggregation. This, in turn, could be explained by the environmental stress caused by the proximity of the RD to Suva City, the major urban centre in the Western South Pacific, outside of Australia and New Zealand. Laucala Bay, which is only 10 km away from the RD, receives high levels of pollution through storm water drains located along the city wall, oil, various chemicals from agricultural runoffs, and sewage effluents from the adjacent Nabukalou Creek^25^. The Suva Port and the Walu Bay Industrial area further contribute to the environmental stress experienced by the biota of Laucala Bay. Lead and other heavy metals that adversely affect the health of coral assemblages are well documented pollutants in the area^26,27^. Furthermore, a few months after the beginning of this study (6^th^ December 2014), a major sewage spill occurred in Suva Harbour, discharging about 200 ls^−1^ of untreated waste water into Laucala Bay. This discharge continued unabated for 18 days, until temporary control measures were implemented leading to a Government Environmental Emergency Declaration prohibiting swimming and fishing in the affected waters. Interestingly, an ongoing analysis of the gut microflora of SHS found that two of the individuals captured in this study on the 12 December 2014 had distinctive profiles dominated by known pathogens; namely *Aeromonas salmonicida* was overrepresented in the microflora of one individual (68.8%) while *Klebsiella pneumonia* was also overrepresented in another one (19.9%). Sharks captured before the spillage had no presence of pathogenic bacteria in their stomachs (Rico C, personal observation). Fishing pressure in the area may also play a role in habitat quality. During the study period, we regularly observed local fishermen setting gillnets overnight and recovering them the next morning. So, if habitat degradation is prevalent in the area, the question arises as why SHS juveniles occur in such quantities in the RD. The apparently most pertinent reason to explain this would be predator avoidance as previously suggested by Duncan and Holland^12^ in Kaneohe Bay.

In general, Yates *et al*.^28^ found that the abundance of SHS in coastal waters was primarily driven by water temperature, salinity and turbidity, with preference for high water temperature, moderate salinity and high turbidity. The environmental profile of the RD mirrors these preferences^29^. However, this study suggested that within the broader RD area there are micro-habitats and spatial preferences of SHS. Neonates and YOY were more likely to be found in sites where waters are shallower (average of 5 m depth), have higher turbidity, warmer temperature, and lower salinity (i.e., sites A, D and E). When considering all sharks, these aforementioned sites as well as F and G show significantly higher CPUE than sites B and C (Fig. 2). The different use of the habitat that emerge from the results may be explained by the presence of a main current between the river mouth and the open ocean at sites B and C, which receives most of the river out flow and energy from incoming ocean waves. This oceanographic process would act to reduce salinity, reduce turbidity, and increase water movement patterns. All of the sites with higher CPUE sit to the easterly side of this current system within the study area. In addition, few studies already suggested that neonates and YOY may stay in shallow waters to avoid predators^6,30^. For instance, based on personal observations, Springer^31^ suggested that neonates and YOY remain in shallow waters to avoid adult feeding grounds.

This study documents the year-round presence of SHS in the RD across the entirety of the study period (Fig. 3). This result is consistent to previous research studies in which neonate, YOY, and juvenile SHS can be found throughout the year^24,28,32,33^. However, in the RD, we noted a significant seasonality to SHS presence (Fig. 3). Further examination of these temporal trends suggests that the parturition period in the RD and/or in its surrounding waters occurs during the wet austral summer season between October and March, with a possible peak between December and February (Table 2). A previous study in Hawaii estimated that the SHS parturition period occurs during the dry boreal spring and summer between May and September^12^, while in Indonesian waters White *et al*.^34^ noted parturition at the beginning of the wet season (between October and November). There are several possible reasons for these differences. Gravid female sharks can regulate the length of their gestation period depending on both their body condition and resource availability^35–37^. In general, the mother’s phenotype directly impacts the phenotype of her young^38,39^, generating a positive correlation between maternal and offspring size^40,41^ and/or the size of the brood^42,43^. This may be especially important for SHS because their newborns are usually in a poor nutritional state^12,44^. More specifically, the surrounding environment (particularly prey availability) as well as condition of the mother likely impacts parturition period length. These factors, as well as the biology of sharks themselves, which allows for fertilisation plasticity^45^ (due to sperm storage), also may allow the timing of parturition to vary between locations. However, critical drivers of parturition period and length are speculative. Considering the multitude of factors that may drive the parturition period and the peak of neonates and YOY in the RD, additional investigations on SHS use of Fiji coastal habitats, and on the role of biotic and environmental factors, are essential to develop a more comprehensive understanding of the biology and ecology of the local SHS population.

On a broader scale, there were two specific events that occurred during the study period which are noteworthy for their potential impact on SHS movement and activity in the RD. Firstly, a Category 5 tropical cyclone passed through the Fiji Islands on 20^th^–21^st^ February, 2016. Sharks of some species have been reported to leave the nursery area during the approach of a tropical storm^46,47^ and so it is plausible that the SHS aggregation of RD may have responded to this severe climatic event in some manner. We recorded lower CPUE in February and March of 2016 as opposed to the previous year (Fig. 5). Secondly, the previously mentioned sewage spillage occurred on 6^th^ December 2014 and resulted in a discharge of about 200 L/s of untreated waste water being released into Laucala Bay (part of the RD) for 18 days until temporary control measures were implemented. The Government Environmental Emergency Declaration prohibited swimming and fishing in the affected waters, including the RD, until 3^rd^ February 2015. We cannot ascertain whether the highest CPUE recorded in this study immediately after the lift of the fishing ban was due to the peak of the parturition season and/or to the decrease in fishing pressure during the period directly prior to our February 2015 sampling trips.

Thirteen percent of SHS were recaptured throughout the period of the study. Compared to fish studies, this rate is quite high. Indeed, for instance, in their summary of tag and recapture data for 33 species of sharks from the National Marine Fisheries Service (NMFS) Cooperative Shark Tagging Program from 1962 to 1993, Kohler *et al*.^48^ showed that the average recapture rate was 3.9%, with a range from 0% for the basking (*Cetorhinus maximus*), finetooth (*Carcharhinus isodon*), smalltail (*Carcharhinus porosus*) and Atlantic angel sharks (*Squatina dumeril*) to 10.9% for the nurse shark (*Ginglymostoma cirratum*). Thus, the high rate of recaptures could be explained by the population dynamics in the RD. Review of the trends in the proportions of SHS exhibiting the various umbilical scars provides support for movement of individuals in and out of the RD, with an apparent great proportion of individuals remaining in the RD. Mortality from fisheries activities is also worth consideration in relation to low recapture rates. During the sampling activities, we regularly saw local fishermen setting overnight gillnets that anecdotally were reported to catch both neonates and YOY SHS. These local catches were either discarded or used for local consumption, and so the level of catches are unknown. Several studies report the threat to sharks posed by artisanal coastal fisheries^49–51^. For instance, Bornatowski *et al*.^50^ reported that large numbers of SHS and smooth hammerhead shark (*Sphyrna zygaena*) were commonly captured over the continental shelf by the artisanal fisheries in Brazil and possibly played a complementary role in the depletion of coastal shark populations. Glaus *et al*.^52^ reported that 81% of Fijian artisanal coastal fishermen that were interviewed (*n* = 253) caught sharks as either bycatch or as a target species. Review of the maps of the interviewees indicate that the RD is an area in which bycatch was reported, yet this does not preclude targeted fishing of sharks by fisherman who were not involved in this study. Further to the discussion above, it is also possible that there are alternative reasons that may explain the recapture rates obtained, such as starvation^12^, tag loss, or aspects of our sampling procedure (e.g. sampling effort).

The integration of the scientific findings gathered from this study into further research efforts and management plans is a necessary next step. An important avenue for future research focus is the systematic collection of CPUE data from a broader geographical area to ascertain the possible boundaries of what might be a primary and/or secondary nursery area^6,53^ – both key critical habitat designations – for SHS in the RD. Furthermore, a fine-scale investigation of SHS individual movement would enhance the understanding of SHS population structure within the study area. A more detailed understanding of environmental and biotic drivers for SHS habitat preference and CPUEs interpretation is also needed. The availability of additional site-specific data such as environmental, physical or oceanographic measurements could be used in the future within a generalised linear mixed model approach to further explore important drivers of SHS presence within the Rewa River. Another limitation of this study concerns the lack of large SHS catches that would allow us to better understand the SHS population structure. A variety of factors could explain this result. For example, the size-selective nature of gillnets is well known, and widely used by fisheries that target sharks, to be an effective tool for managing the size composition of catches^54^. In this study, we used gillnets with a small mesh size (~10 cm) in order to catch neonates and YOY, explaining the lack of adults catches. However, although no adults were caught, several times big holes in the net were observed, suggesting large sharks going through the net. In addition, we also observed large shark dorsal fins before sunset in several occasions. The spatial limitation of the sampling area could be another factor that did not allow large SHS catches. A recent study demonstrated that SHS populations are segregated by sex and size. Chin, *et al*.^55^ showed that Australian populations are dominated by juveniles and small adult males, while Indonesian and PNG populations include large adult females. In general, adults SHS are known to occur in open ocean habitats and make long distance migrations^56,57^. Consequently, the sampling area which encompass an area of only 5.5 km^2^ in a river delta also decreases the probability to catch any large SHS.

### Fisheries management implications

The multispecies fishery operating in the RD is small-scale and includes both subsistence and commercial fishing. Several fishing gears are used^58^, but captures of SHS have been anecdotally reported only by fishermen using gillnets. SHS are not targeted, but the individuals incidentally entangled in the gillnets are believed to most often be kept; likely for subsistence purposes. With classic market surveys being inappropriate, we shifted the attention to individual interviews, yet due to the sensitive nature of the topic we deemed the information to be somewhat unreliable.

Like in most developing countries, the coastal subsistence and commercial fisheries are data-poor^59^. Onboard observers only operate on a small fraction of Fiji’s industrial offshore fishery^17,18^, while the coastal fisheries are virtually unmonitored^52,60^, so that the combined capture of sharks is greatly underreported and/or underestimated^61^. As involvement of local leaders has proved to be effective for collecting reliable fisheries data from local fishermen in other locations^62^, we suggest that traditional and religious leaders be involved to facilitate data collection on the level of exploitation in Fiji. This will allow reliable estimates of the number of SHS annually caught in the RD, and would serve as a baseline to assess the effectiveness of any future management measure of the SHS aggregation in the RD by conducing regular monitoring.

The use of gillnets to fish for teleosts in the RD appears to be the only direct threat that we noticed during this study. While anecdotal accounts of shark catches cannot ascertain a reliable mortality rate, the interviewed fishermen reported up to 50 YOY SHS deaths per week/fishermen as bycatch at the beginning of the austral summer. The most promising management approach to reduce the fishing mortality of neonate and YOY SHS in the RD is a seasonal fishing gear closure. In particular, we strongly encourage a ban of gillnet fishing during the parturition period, which this study identified as the months of the austral summer, from October to April. This management tool has already been adopted in other countries^63–65^. Conservation costs of this management should be offset to avoid negative consequences on the livelihood of those that fish for subsistence, or on those whose only income is from fishing with gillnets, for example by redirecting wild captures towards aquaculture.

Results from this study were presented to the Rewa Provincial Council and Fiji Fisheries Department and in 2017 the Chiefs of Rewa declared their willingness to establish a Marine Protected Area (MPA)^66^ in the RD. However, the implementation and management of such an area will require extended consultation with the many relevant stakeholders.

A hopeful approach would be to consider the framework of marine management set out under the growing network of locally managed marine areas (LMMA) in Fiji, whose success is based on the involvement of local villagers in development and management processes, from the discussion of their priorities before establishing a new area, to villagers commitment towards the agreed targets and measures^67,68^. Enhancing coastal fisheries management by empowering local communities is envisaged for the whole Pacific region^69^, with both coastal communities and marine resources benefitting from better monitoring and compliance. During the 2017 United Nations Ocean Conference, Fiji undertook a voluntary commitment to have all customary marine areas managed by local communities by 2025, to achieve sustainable use of the marine resources (https://oceanconference.un.org). Hence, an excellent step forward to protect SHS in the RD and honour this national commitment would be to include the RD as a locally managed LMMA within the Fiji LMMA network.

## Methods

### Sampling site

This study focused on the RD (178.55°E, 18.15°S, Fig. 1) on Viti Levu, Fiji. The delta is the largest local fluvial system and originates from the Rewa River, the longest insular river. Although the RD is approximately 9 km^2^, the sampling sites only encompassed an area of about 5.5 km^2^. The RD is characterised by strong currents and high wave actions as a result of the collision between the river runoff and the incoming ocean waves/tides via the reef channel. This interaction gives the RD estuarine habitat conditions such as large fluctuating salinities, a freshwater layer, high turbidity and tidal waves^29,70^, which make 40% of the RD inaccessible for sampling. The RD was chosen as the focal point for this study because it is recognised through local ecological knowledge that the Rewa Delta and River are areas of aggregation for juvenile hammerhead and bulls sharks. Furthermore, two studies^19,20^ pointed out that the anecdotal accounts from locals were probably correct. Finally, during preliminary surveys, we deployed the net up to 10 times east and west of the RD and had no catches of SHS, while in the Rewa Delta we captured 23 individuals in the first 10 hours of work, confirming that the focal area was within the Delta and not in the surrounding areas. A local fishermen with 45 years of fishing experience in the area guided the fieldwork throughout the study, and consistently informed us with great accuracy of the places where sharks occurred.

### Sampling design

We divided the study area into seven equally sized quadrats of approximately 1 km^2^. In the month of September 2014, a pilot survey was conducted with a total of 42 gillnets soaked for one hour each without a clear spatial sampling scheme by randomly deploying the net in six sites per quadrant, out of 18 equidistant points scattered within each quadrat. This pilot survey was conducted in order to test the sampling methodology and procedures, and to identify suitable areas for sampling. Suitable areas were determined by accessibility, exposure to wind and swells and strength of currents, as this was a determining factor in the success of subsequent deployments. After the pilot study, several sites were identified as sites of interest for long-term monitoring using the above criteria (Fig. 1). The area covered by the sites of interest ranged from the near shore (north) to the deep/reef channel areas, and most of the sheltered area. Each site was sampled at least 3 times per month. Surveys were carried out for a total of 19 months (from September 2014 to March 2016, inclusive). No field work was carried out during the month of January 2015 as a result of a ban on fishing in Suva coastal waters due to a sewage spill.

### Sampling methods

Sampling was conducted under a research permit provided by the Rewa Provincial Council and the Fiji Government Department of Fisheries. All handling procedures of live SHS specimens were approved under the “Animal Ethics Committee” section of The University of the South Pacific (USP) Research Committee and performed in accordance with relevant guidelines and regulations. Fishery independent surveys were carried out using bottom set gillnets^28,71,72^ deployed between sunset (around 18:00) and midnight^12,24^. A gillnet of ~10 cm mesh size was chosen as they are highly selective for small sharks and have been used in most scientific field studies of this nature^73^. The gillnet was made of nylon monofilament, 9 ply thick, and was 3 m high and 100 m long. A gillnet survey consisted of soaking the net at a particular site for 1 hour, with 15 minute checking intervals to minimise mortality and increase the survival probability of the released SHS^28,33,74,75^.

### Shark processing

For each individual shark the species was identified according to Last and Stevens^76^, and sex and umbilical scar status were noted. The umbilical scar was categorised based on healing stage (e.g. open, semi-healed, healed, well-healed)^12^. Opened and semi-healed umbilical scars characterise the neonate period. The neonate period lasts for approximately 15 days, during which time the umbilical scar becomes closed and SHS individuals may reach a maximum of 61 cm in TL. YOY are identified by a healed or well-healed umbilical scar. The total length (TL) of each SHS was taken to the nearest centimetre^13^ using a measuring board designed to accommodate the particular shape of the SHS head. To mark the released sharks, PIT (Passive Integrated Transponder) tags were injected directly beneath the skin tissue at the base of the first dorsal fin^77^. The PIT tags were of dimensions 2 × 12 mm and emitted a frequency of 134.2 kHz (KingDoes RFID Technology, model ISO FDX-B). Recapture identification was carried out by scanning all sharks brought aboard. At first capture, two biopsy tissue samples of about 5 mm each were taken from the pelvic fins of each SHS for future genetic analyses. It was noticed from recaptured individuals that the biopsy wound was healed and regenerated within two to three weeks.

### Data analysis

The population structure was assessed by comparing the CPUE of sharks at sites of interest. CPUE was standardised by summing the total number of sharks caught and dividing by 100 m (length) × 3 m (high) of net over a one hour time period for each net set. CPUE was calculated for the total number of sharks and for neonates only (i.e., individuals with open and semi-healed umbilical scars). Normality and variance of the CPUE data indicated that data distribution was not normal nor homogeneous, and so non-parametric tests (Multiple Comparison Procedures for Unbalanced One-Way Factorial Designs)^78^ were performed. These analyses were undertaken using the R package nparcomp^79^ at an α-level of 0.05.

The population structure over time was then examined through direct summation of database records. The total number of recaptures, as well as length of time between recaptures, was collated. We also assessed monthly patterns in CPUE across the study period. Non-parametric comparison tests were used to detect significant differences as discussed previously.

Trends in the relative counts of sharks with open, semi-healed and healed umbilical scars were examined on a monthly basis using chi-square analyses. Sharks with well-healed umbilical scars were excluded from this analysis due to the small sample size, which did not meet the minimum count requirement per group. Standardised Pearson residuals were inspected to assess whether any of the given scar statuses occurred at higher or lower than expected proportions in any given month than would be anticipated if there were no clear parturition period for SHS in the RD.

Total length data of SHS collected in this study were also summarised to provide a baseline for comparison with future and other studies. Descriptive statistics (i.e., minimum, maximum, and median TL) were provided for the four different scar categories, sexes, and sites. Differences in TL for the four umbilical scar categories were examined using non-parametric analyses (see above)^78^. Monthly growth rates between recaptures were estimated separately for males and females by averaging (±SD) the difference in length between the first and the last capture.

## Electronic supplementary material

Supplementary Information

## References

[1] Worm B (2013). Global catches, exploitation rates, and rebuilding options for sharks. Mar. Pol..

[2] Clarke SC, Harley SJ, Hoyle SD, Rice JS (2013). Population trends in Pacific Oceanic Sharks and the utility of regulations on shark finning. Conserv. Biol..

[3] Baum JK (2003). Collapse and Conservation of Shark Populations in the Northwest Atlantic. Science.

[4] Walker T (1998). Can shark resources be harvested sustainably? A question revisited with a review of shark fisheries. Mar. Freshw. Res..

[5] Simpfendorfer CA, Dulvy NK (2017). Bright spots of sustainable shark fishing. Curr. Biol..

[6] Heupel MR, Carlson JK, Simpfendorfer CA (2007). Shark nursery areas: concepts, definition, characterization and assumptions. Mar. Ecol.-Prog. Ser..

[7] Karl SA, Castro ALF, Lopez JA, Charvet P, Burgess GH (2011). Phylogeography and conservation of the bull shark (*Carcharhinus leucas*) inferred from mitochondrial and microsatellite DNA. Cons. Gen..

[8] Chapman DD, Feldheim KA, Papastamatiou YP, Hueter RE (2015). There and Back Again: A Review of Residency and Return Migrations in Sharks, with Implications for Population Structure and Management. Annu. Rev. Mar. Sci..

[9] Keeney DB, Heupel MR, Hueter RE, Heist EJ (2005). Microsatellite and mitochondrial DNA analyses of the genetic structure of blacktip shark (*Carcharhinus limbatus*) nurseries in the northwestern Atlantic, Gulf of Mexico, and Caribbean Sea. Mol. Ecol..

[10] Tillett J, Meekan MG, Field IC, Thorburn DC, Ovenden JR (2012). Evidence for reproductive philopatry in the bull shark *Carcharhinus leucas*. J. Fish. Biol..

[11] Feldheim KA (2014). Two decades of genetic profiling yields first evidence of natal philopatry and long-term fidelity to parturition sites in sharks. Mol. Ecol..

[12] Duncan K, Holland K (2006). Habitat use, growth rates and dispersal patterns of juvenile scalloped hammerhead sharks *Sphyrna lewini* in a nursery habitat. Mar. Ecol.-Prog. Ser..

[13] Compagno, L. J. V. *FAO species catalogue. Vol.4. Sharks of the world. An annotated and illustrated catalogue of shark species known to date. Part 1. Hexanchiformes to Lamniformes*., Vol. 4 249 (FAO Fish Synop., 1984).

[14] Compagno, L. J. V. *FAO species catalogue. Vol.4. Sharks of the world. An annotated and illustrated catalogue of shark species known to date. Part 2. Carcharhiniformes*., Vol. 4 251–655 (FAO Fish Synop., 1984).

[15] Baum, J. *et al*. *Sphyrna lewini* (Northwest and Western Central Atlantic subpopulation). The IUCN Red List of Threatened Species 2007: e. T165293A6000960. 10.2305/IUCN.UK.2007.RLTS.T165293A6000960.en (2007).

[16] CITES. cites.org. [ONLINE]Available at: https://cites.org/eng/app/appendices.php. [Accessed 04 February 16], 2015).

[17] Piovano, S. & Gilman, E. Elasmobranch captures in the Fijian pelagic longline fishery. *Aquat. Conserv.-Mar. Freshw. Ecosyst*. **27**, 381–393, 10.1002/aqc.2666 (2017).

[18] Gilman E (2008). Shark interactions in pelagic longline fisheries. Mar. Pol..

[19] Rasalato E, Maginnity V, Brunnschweiler JM (2010). Using local ecological knowledge to identify shark river habitats in Fiji (South Pacific). Environ. Conserv..

[20] Brown KT, Seeto J, Lal MM, Miller CE (2016). Discovery of an important aggregation area for endangered scalloped hammerhead sharks, *Sphyrna lewini*, in the Rewa River estuary, Fiji Islands. Pac. Conserv. Biol..

[21] Branstetter S (1987). Age growth and reproductive biology of the silky shark, *Carcharinus falciformis*, and the scalloped hammerhead, *Sphyrna lewini*, from the northwestern Gulf of Mexico. Environ. Biol. Fishes.

[22] Holland KN, Wetherbee BM, Peterson JD, Lowe CG (1993). Movements and Distribution of Hammerhead Shark Pups on Their Natal Grounds. Copeia.

[23] Harry AV, Macbeth WG, Gutteridge AN, Simpfendorfer CA (2011). The life histories of endangered hammerhead sharks (Carcharhiniformes, Sphyrnidae) from the east coast of Australia. J. Fish. Biol..

[24] Clarke, T. The ecology of the Scalloped Hammerhead Shark, *Sphyrna lewini* in Hawaii. *Pac. Sci*. **25**, 133–144, http://hdl.handle.net/10125/4191 (1971).

[25] Naidu SD, Morrison RJ (1994). Contamination of Suva Harbour, Fiji. Marine Pollution Bulletin.

[26] Prakash R, Jokhan AD (2013). Photosynthetic rate and biochemical composition of green algae Ulva flexuosa (Wulfen) J. Agardh as potential indicators of environmental stress in the intertidal zones. The South Pacific Journal of Natural and Applied Sciences.

[27] Tabudravu J, Gangaiya P, Sotheeswaran S, South G (2002). Enteromorpha flexuosa (Wulfen) J. Agardh (Chlorophyta: Ulvales)–evaluation as an indicator of heavy metal contamination in a tropical estuary. Environmental monitoring and assessment.

[28] Yates PM, Heupel MR, Tobin AJ, Simpfendorfer CA (2015). Ecological Drivers of Shark Distributions along a Tropical Coastline. PLoS One.

[29] Singh A, Aung T (2008). Salinity, Temperature and Turbidity Structure in the Suva Lagoon, Fiji. Am. J. Environ. Sci.

[30] Duncan K, Martin AP, Bowen BW, HG DEC (2006). Global phylogeography of the scalloped hammerhead shark (*Sphyrna lewini*). Mol Ecol.

[31] Springer, S. In *Sharks, skates and rays* (eds Gilbert, P. W., Mathewson, R. F., & Rall, D. P.) 149–174 (John Hopkins, 1967).

[32] DeAngelis BM, McCandless CT, Nancy EK, Recksiek CW, Skomal GB (2008). First characterization of shark nursery habitat in the United States Virgin Islands: evidence of habitat partitioning by two shark species. Mar. Ecol.-Prog. Ser..

[33] Castro J (1993). The shark nursery of Bulls Bay, South Carolina, with a review of the shark nurseries of the southeastern coast of the United States. Environ. Biol. Fishes.

[34] White WT, Bartron C, Potter IC (2008). Catch composition and reproductive biology of *Sphyrna lewini* (Griffith & Smith) (Carcharhiniformes, Sphyrnidae) in Indonesian waters. J. Fish. Biol..

[35] Hussey NE (2010). Maternal investment and size-specific reproductive output in carcharhinid sharks. J. Anim. Ecol..

[36] Kiltie RA (1982). Intraspecific Variation in the Mammalian Gestation Period. J. Mammal..

[37] Mysterud A, Roed KH, Holand O, Yoccoz NG, Nieminen M (2009). Age-related gestation length adjustment in a large iteroparous mammal at northern latitude. J. Anim. Ecol.

[38] Bernardo J (1996). Maternal Effects in Animal Ecology. Am. Zool..

[39] Mousseau, T. A. & Fox, C. W. *Maternal effects as adaptations*. 400 (Oxford University Press, 1998).

[40] Côté SD, Festa-Bianchet M (2001). Reproductive success in female mountain goats: the influence of age and social rank. Anim. Behav..

[41] Bridget SG, Mark IM (2005). Maternal and paternal effects determine size, growth and performance in larvae of a tropical reef fish. Mar. Ecol.-Prog. Ser..

[42] Sogard SM, Berkeley SA, Fisher R (2008). Maternal effects in rockfishes *Sebastes spp*.: a comparison among species. Mar. Ecol.-Prog. Ser..

[43] Morris DW (1996). State dependent life histories. Mountford’s hypothesis, and the evolution of brood size. J. Anim. Ecol..

[44] Lowe CG (2002). Bioenergetics of free-ranging juvenile scalloped hammerhead sharks (*Sphyrna lewini)* in Kaneohe Bay,Oahu, HI. J. Exp. Mar. Biol. Ecol..

[45] Pratt HL (1993). The storage of spermatozoa in the oviducal glands of western North Atlantic sharks. Environ. Biol. Fishes.

[46] Udyawer V, Chin A, Knip DM, Simpfendorfer CA, Heupel MR (2013). Variable response of coastal sharks to severe tropical storms: environmental cues and changes in space use. Mar. Ecol.-Prog. Ser..

[47] Heupel MR, Simpfendorfer CA, Hueter RE (2003). Running before the storm: blacktip sharks respond to falling barometric pressure associated with Tropical Storm Gabrielle. J. Fish. Biol..

[48] Kohler NE, Casey JG, Turner PA (1998). NMFS cooperative shark tagging program, 1962-93: an atlas of shark tag and recapture data. Mar Fish Rev.

[49] Vianna GMS, Meekan MG, Ruppert JLW, Bornovski TH, Meeuwig JJ (2016). Indicators of fishing mortality on reef-shark populations in the world’s first shark sanctuary: the need for surveillance and enforcement. Coral Reefs.

[50] Bornatowski H, Braga RR, Vitule JRS (2014). Threats to sharks in a developing country: The need for effective simple conservation measures. Natureza & Conservação.

[51] Quintanilla S (2015). Conservation Genetics of the Scalloped Hammerhead Shark in the Pacific Coast of Colombia. J. Hered..

[52] Glaus KBJ, Adrian-Kalchhauser I, Burkhardt-Holm P, White WT, Brunnschweiler JM (2015). Characteristics of the shark fisheries of Fiji. Sci Rep.

[53] Bass, A. J. In *Sensory biology of sharks, skates and rays* (ed Hodgson & Mathewson) 545–594 (1978).

[54] McAuley RB, Simpfendorfer CA, Wright IW (2007). Gillnet mesh selectivity of the sandbar shark (Carcharhinus plumbeus): implications for fisheries management. Ices Journal of Marine Science.

[55] Chin, A. *et al*. Crossing lines: a multidisciplinary framework for assessing connectivity of hammerhead sharks across jurisdictional boundaries. *Sci Rep* **7**, 10.1038/srep46061 (2017). 10.1038/srep46061 PMC5399444 28429742

[56] Diemer KM, Mann BQ, Hussey NE (2011). Distribution and movement of scalloped hammerhead *Sphryna lewini* and smooth hammerhead *Sphyrna zygaena* sharks along the east coast of southern Africa. Afr. J. Mar. Sci..

[57] Ketchum JT (2014). Inter-island movements of scalloped hammerhead sharks (Sphyrna lewini) and seasonal connectivity in a marine protected area of the eastern tropical Pacific. Mar. Biol..

[58] Tuiwawa, M. V., Pene, S. & Tuiwawa, S. A Rapid Biodiversity Assessment, Socioeconomic Study and Archaeological Survey of the Rewa River Mangroves, Viti Levu, Fiji. *Report for the Fiji Department of Environment ‘Mangrove Ecosystems for Climate Change and Livelihood’ (MESCAL) Programme*. (2013).

[59] Gillett, R., Lewis, A. & Cartwright, I. Coastal Fisheries in Fiji: Resources, Issues, and Enhancing the Role of the Fisheries Department. *Report for David and Lucille Packard Foundation*. (2014).

[60] Lack, M. & Meere, F. Pacific Islands regional plan of action for sharks: Guidance for Pacific Island countries and territories on the conservation and management of sharks. *Report for FFA, SPC, SPREP/PROE*. (2009).

[61] Juncker, M., Robert, M. & Clua, E. Coastal shark fisheries in the Pacific: a brief overview of current knowledge. *Report for the CRISP Programm*e. (2006).

[62] Piovano S, Basciano G, Swimmer Y, Giacoma C (2012). Evaluation of a bycatch reduction technology by fishermen: A case study from Sicily. Mar. Pol..

[63] Williams H, Schaap A (1992). Preliminary results of a study into the incidental mortality of sharks in Gill-nets in two Tasmanian Shark Nursery Areas. Mar. Freshw. Res..

[64] Walker, T. I. (ed. FAO Fisheries Technical Paper No. 378/2. Rome) 480–514 (1999).

[65] Simpfendorfer, C. A. Management of shark fisheries in Western Australia. In Shotton, R. (ed.). Case studies of the management of elasmobranch fisheries. *FAO Fisheries Technical Paper No. 378/1. Rome*, 425–455 (1999).

[66] Susu, A. Chiefs declare Rewa River marine protected. *Fiji Times*, http://www.fijitimes.com/story.aspx?id=403423 (2017).

[67] Jupiter SD, Egli DP (2011). Ecosystem-Based Management in Fiji: Successes and Challenges after Five Years of Implementation. Journal of Marine Biology.

[68] Weeks R, Jupiter SD (2013). Adaptive Comanagement of a Marine Protected Area Network in Fiji. Conserv. Biol..

[69] Community, S. o. t. P. A new song for coastal fisheries – pathways to change: The Noumea strategy. *SPC Npumea, New Caledonia*. (2015).

[70] Mohammed SWC, Coppard SE (2008). Ecology and distribution of soft-sediment benthic communities off Viti Levu (Fiji). Mar. Ecol.-Prog. Ser..

[71] Froeschke J, Stunz G, Sterba-Boatwright B, Wildhaber M (2010). An empirical test of the shark nursery area concept in Texas bays using a long-term fisheries-independent data set. Aquat. Ecol..

[72] Merson R, Pratt H (2001). Distribution, Movements and Growth of Young Sandbar Sharks, *Carcharhinus Plumbeus*, in the Nursery Grounds of Delaware Bay. Environ. Biol. Fishes.

[73] Hueter RE, Charles AM, John PT, John MH, Daniel AH (2006). Assessing mortality fo released or discarded fish using a logistic model of relative survival derived from tagging data. Trans. Am. Fish. Soc..

[74] Ulrich GF (2007). Habitat utilization, relative abundance, and seasonality of sharks in the esturine and nearshore waters of South Carolina. Am. Fish. Soc. Symp..

[75] Adams, D. H. & Paperno, R. In *Shark nursery grounds of the Gulf of Mexico and the east coast of the United States*. Vol. 50 (eds McCandless, C. T., Kohler, N. E., & Pratt, H. L.) 165–174 (American Fisheries Society, 2007).

[76] Last, P. R. & Stevens, J. D. *Sharks and rays of Australia*., (CSIRO, 1994).

[77] Gibbons WJ, Andrews KM (2004). PIT Tagging: Simple Technology at Its Best. BioScience.

[78] Gao X, Alvo M, Chen J, Li G (2008). Nonparametric multiple comparison procedures for unbalanced one-way factorial designs. J. Stat. Plan. Infer..

[79] Konietschke F, Placzek M, Schaarschmidt F, Hothorn L (2015). A. nparcomp: An R Software Package for Nonparametric Multiple Comparisons and Simultaneous Confidence Intervals. J. Stat. Softw..

[80] R Development Core Team. R: A language and environment for statistical computing. *R Foundation for Statistical Computing, Vienna, Austria*, http://www.R-project.org (2008).

