# Supplementary material for: Fisheries-independent surveys identify critical habitats for young scalloped hammerhead sharks (Sphyrna lewini) in the Rewa Delta, Fiji
Source: Sci Rep. 2017 Dec 8;7:17273. doi: 10.1038/s41598-017-17152-0 (PMC5722814; doi:10.1038/s41598-017-17152-0)
Supplement: Supplementary file 1 — Supplementary Information [file 41598_2017_17152_MOESM1_ESM.docx]

Fisheries-independent surveys identify critical habitats for young scalloped hammerhead sharks (*Sphyrna lewini*) in the Rewa Delta, Fiji

Amandine D. Marie, Cara Miller, Celso Cawich, Susanna Piovano, Ciro Rico

**Supplementary Table S1**: Median, minimum and maximum total length (cm), and number of scalloped hammerhead sharks considering the sex of the individuals, their umbilical scar status and the sampling site where they were caught.

| **Parameter** | **Median TL** | **Minimum TL** | **Maximum TL** | **Number of individuals** |
| --- | --- | --- | --- | --- |
|  | | | | |
| **Sex** | | | | |
|  | | | | |
| **Male** | 55.0 | 42.0 | 86.0 | 552 |
| **Female** | 53.0 | 35.0 | 116.5 | 476 |
|  | | | | |
| **Umbilical scar status** | | | | |
|  | | | | |
| **Open** | 50.4 | 46.0 | 56.0 | 47 |
| **Semi-healed** | 51.0 | 35.0 | 61.0 | 240 |
| **Healed** | 55.5 | 39.5 | 86.0 | 734 |
| **Well-healed** | 71.5 | 69.5 | 80.0 | 6 |
|  | | | | |
| **Sampling site** | | | | |
|  | | | | |
| **A** | 53.0 | 39.5 | 75.0 | 151 |
| **B** | 53.5 | 48.5 | 78.0 | 20 |
| **C** | 53.0 | 48.0 | 70.0 | 18 |
| **D** | 53.0 | 35.0 | 80.0 | 425 |
| **E** | 53.1 | 45.0 | 82.0 | 166 |
| **F** | 58.0 | 47.5 | 116.5 | 168 |
| **G** | 58.4 | 38.5 | 86.0 | 80 |
